# Case management and care expertise as a prevention approach for adults with intellectual disabilities (FaPP-MgB): study protocol for a randomized-controlled trial

**DOI:** 10.1186/s13063-023-07155-w

**Published:** 2023-02-23

**Authors:** Stephan Nadolny, Dirk Bruland, Marie Grunwald, Annika Gröndahl, Jessica Grammatico, Miriam Tariba Richter, Christian Grebe, Änne-Dörte Latteck

**Affiliations:** 1 Institute for Educational and Health-Care Research in the Health Sector, Bielefeld University of Applied Sciences, Interaktion 1, 33619 Bielefeld, Germany; 2 Institute for History and Ethics of Medicine, Interdisciplinary Center for Health Sciences, Martin Luther University Halle-Wittenberg, Magdeburger Str. 8, 06112 Halle, Germany; 3 Institute for Management and Technology, Osnabrück University of Applied Sciences, Kaiserstraße 10C, 49809 Lingen, Germany; 4 Competence Center for Health, Hamburg University of Applied Sciences, Alexanderstraße 1, 20099 Hamburg, Germany

**Keywords:** Advanced nursing practice, Nursing, Case management, Prevention, Health promotion, Intellectual disabilities, Randomized-controlled trial, Germany

## Abstract

**Background:**

Adults with intellectual disabilities have a higher prevalence of unhealthy eating habits, stress, low levels of mobility, and comparable drug consumption as the general population. Consequently, they suffer from several chronic diseases earlier and more often, but there are fewer prevention and health promotion services including this population. The goal of this study is to determine if an advanced practice nursing approach in the community with home visits is an effective way to improve the health status of adults with intellectual disabilities.

**Methods:**

We will conduct a randomized-controlled trial with waiting list design in Hamburg, Germany. Inclusion criteria are diagnosis ICD F70-F79 and exclusion criteria are care level > 3 according to the German Social Code XI or being at the end-of-life. Participants will be block randomized. The intervention consists of advanced practice nurses performing case management, social space analysis, prevention planning, and counseling through four outreach home visits on nutrition, mobility, addiction, and stress. Comparison is usual care. The primary outcome is health status (WHODAS) after 12 months. Secondary outcomes are health-related quality of life (EQ-5D) and resilience (RS-11) after 6 and 12 months. The calculated sample size is 256 with an estimated dropout of 30%. Raters and analysts will be blinded. Analysis will be performed using ANCOVAs.

**Discussion:**

By providing case management and utilizing their nursing expertise, advanced practice nurses will provide valuable input and guidance on prevention and health promotion for people with intellectual disabilities. They will close the gap between health and social care, which is prominent in Germany, through cooperation between the existing care sectors.

The findings will be disseminated in peer-reviewed journals and presented at national and international conferences.

**Trial registration:**

German Clinical Trials Register, DRKS00028771, registered 4 July 2022, Universal Trial Number: U1111-1277–0595.

## Introduction

People with intellectual disabilities^1^ account for approximately 1% of the population depending on method of measurement and context [1, 2]. They have higher risk of certain (chronic) diseases depending on context, e.g. asthma, diabetes, epilepsy, obesity, and osteoporosis as compared to the general population [3, 4, 5]. They seem to show high levels of sedentary behavior [6] and low levels of physical activity [7]. Additionally, people with intellectual disabilities are more susceptible to unhealthy eating habits [8] as well as stress [9] throughout their life and show similar levels of tobacco and alcohol consumption as the general population [10]. However, they seem to be more vulnerable to the side effects of drug consumption [11]. Therefore, they have an elevated mortality which could have been mitigated by healthcare interventions [12, 13, 14, 15].

As any other group, people with intellectual disabilities could make use of more health services with regard to prevention and health promotion. However, there are several factors that, among others, result in lower rates of received prevention and health promotion interventions [16]. First, programs enrolling people with intellectual disabilities are rare (in Germany) since professionals often do not have the competence to deal with these clients. Second, people with intellectual disabilities may have different communication patterns [17]. Third, due to a lack of data, we assume that a considerable amount of people has a lower health literacy like the general population [18, 19], and therefore, identifying suitable information regarding the topic of prevention and health promotion is difficult [20]. Fourth, the presented information (written or verbal) is often not understandable [20]. Fifth, people with intellectual disabilities might also have lower resilience [21] while facing more adversity throughout their life than the general population [22].

To address these issues, specialized care and support systems need to be established, which focus on the (social) environment as well as individual aspects [23, 24]. This implicates that some form of case management could be helpful improving the situation for people with intellectual disabilities. However, the German nursing context differs from most other countries in terms of graduation and roles.

The education of the majority of nurses takes place at vocational (nursing) schools. The opportunity to study for a primary qualification in nursing only exists for about 18 years with a considerable increase around 2010 due to an initiative for model study programs [25]. The academic qualification in nursing management, science, and education exists since the early 1990s [26]. Overall, about 2–3.2% of nurses have an academic qualification, most of them on a bachelor’s level [27]. Additionally, the German healthcare system is very physician-centered, especially with regard to prescriptions of medication and medical aids or referral to other healthcare practitioners. Consequently, Germany lags significantly behind the international developments of nursing care of other countries. Therefore, advanced nursing practice (ANP) is only seldomly implemented in Germany. There are several hospitals using advanced practice nurses (APN) expertise, but no systematic approach exists in Germany [28]. In the community setting, it is even more rare. Pilot projects in Germany most recently successfully implemented school nurses [29].

In Germany, the profession of nursing does not have a longstanding tradition in caring for people with intellectual disabilities. This field has mostly been worked by practitioners from curative education, social work, or social pedagogy. Only in recent years, in the course of the reform of the German Teilhabegesetz (Participation Act), it has been advocated to bring more health professionals into this field, as the health problems of this population group have not been given sufficient attention. We believe that APNs will be able to combine the elements of case management, prevention, health promotion, and working on the (social) environment to improve the care and health of people with intellectual disabilities as indicated by a recent systematic review on the role and key activities of APNs [30]. The project “Case management and care expertise as a prevention approach for adults with intellectual disabilities” takes up this demand and develops a prevention offer for people with intellectual disabilities from the APN understanding.

Regarding the effectiveness of case management, up to this date, there still remains only limited evidence [31, 32, 33, 34, 35, 36]. The effects of case management are not that easily measured with quantitative studies alone, because it is a complex intervention often working between the different sectors of care. Especially the impact on health outcomes might often be mitigated by other factors [37]. Nonetheless, it might help to reduce hospital admissions [31, 33, 34, 35], although there also exists contrasting evidence [36, 38]. Moreover, it can also possibly have an impact on self-care and self-management as well as the communication between healthcare professionals and the patients and the caregivers [31]. This strongly varies between populations and contexts. On the other hand, qualitative studies highlight, among other things, the improvement of access to healthcare as a door opener through coordination as well as advocacy, improved understanding of health, continuous care due to individualized care plans, networking, and emotional support [39, 40]. Considerable barriers for case management are time constraints, lack of willingness to work interdisciplinary by other health professionals, lack of training, and confusion regarding the case manager’s role [40, 41].

With respect to people with intellectual disabilities, only very few studies have been conducted on case management itself as an intervention so far [42, 43]. However, case management is usually a part of nursing itself and is included in interventions regarding prevention and health promotion for this population. The existing evidence on the effects of prevention and health promotion on adults with intellectual disabilities is sparse, although there are 17 reviews on the topic. Most of them include only a few studies. The biggest body of evidence exists for weight management and lifestyle changes with 61 studies overall [5].

Studies focusing specifically on the population at hand identified the effectiveness of physical activity interventions on maintaining and improving physical activity. Additionally, specific exercises might improve the people’s gait. However, the effect of interventions of activity and weight management alone on for example the BMI or food intake are mixed [5, 44]. The combination of interventions focusing on activity and nutrition seem to be more promising [5]. The impact of specific interventions to reduce substance use in any form or improve stress levels do not show conclusive results [5, 11, 45].

Our study will provide further insight into nurse-led case management regarding people with intellectual disabilities and ANP in Germany.

## Methods

### Objectives

The goal of the study is to (a) test the effectiveness of the ANP intervention on the health status, resilience, and quality of life of people with intellectual disabilities, (b) evaluate the process of the intervention, and (c) perform a health economic evaluation. Here, we will report on (a) and (b) only.

### Design

The study design is a randomized-controlled trial (RCT) with waiting list and superiority framework. People being in the control group (CG) will receive the intervention after completion of data collection after 12 months. It will be conducted from January 2022 to December 2024. The study is accompanied by a mixed-methods process evaluation consisting of quantitative structured interviews as well as qualitative interviews focusing on the process as well as experiences of different stakeholders (APNs, clients, caregivers, care providers in the community) as well as an inclusive research group. In the latter, people with intellectual disabilities will act as co-researchers and conceptualize their own part in the evaluation. We will report on the RCT only, because the conceptualization is finalized. The flow of participants and data collection is shown in Table 1.

**Table 1:**
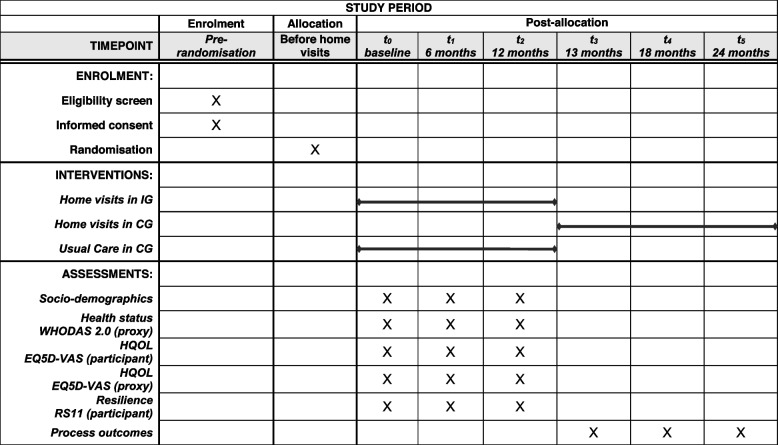
SPIRIT - Phases of trial and data collection

*IG* Intervention Group, *CG* Control Group, *HQOL* Health-related quality of life

### Participants and randomization

The study will be conducted in all seven districts of Hamburg, Germany. Therefore, participants can live in the community setting as well as in more residential care settings. A list of our cooperation partners can be found in the trial register. Inclusion criteria are a diagnosis of ICD-11 F70-F79 and being ≥ 18 years. Exclusion criteria are having a care level > 3 according to the German Social Code XI (which corresponds to severe impairments of independence or abilities), as well as being at the end-of-life.

Recruitment started in July 2022. Study participants are recruited through (a) local information events with a presentation and room for questions in various contexts, e.g., in residential institutions caring for people with intellectual disabilities, residential living groups, housing associations, cultural street festivals, and (b) mailings by the German health insurance funds AOK Rheinland/Hamburg and Mobilkrankenkasse which includes advertisements and a statement of support for the study as well as contact details of the coordinator for the APNs at the Evangelische Stiftung Alsterdorf (ESA).

Randomization is going to be conducted with the R-Package randomizeR [46] with randomly permuted blocks in size of two, four, or six [47]. The person randomizing the participants at the Bielefeld University of Applied Sciences (BUAS) is also responsible for scheduling the raters for data collection. She is going to send the list of randomized pseudonyms to the person doing the appointment coordination for the APNs at ESA. Afterwards, BUAS will inform the participants and if applicable their legal guardians in the CG and intervention group (IG) about the result of the randomization and will make appointments for data collection, while the ESA is doing calls for scheduling the APN visits. The person at BUAS is the only person that has access to the randomization procedure and keeps the list of randomizations password protected.

Group assignment will be blinded for raters as well as the analyst. Participants as well as the APNs cannot be blinded due to the nature of the intervention. The clients as well as their caregivers are instructed not to talk about the intervention before each data collection; however, it can be expected that some clients may tell the raters about the APNs visits.

### Intervention

Nurses on a master’s level (APN) are supposed to work as case managers and utilize their advanced nursing expertise. They are employed at the ESA for this study. They will take on all basic functions of case management: gatekeeping, brokering, and advocacy [48]. The intervention focuses on prevention and healthcare promotion for people with intellectual disabilities. It targets four areas of prevention, because the legally determined guideline for prevention in Germany identifies these as aspects to be dealt with in the foreground. Additionally, problems in this area are quite prevalent in people with intellectual disabilities:

- Mobility
- Nutrition
- Stress
- Addiction

We do not emphasize the differentiation between primary, secondary, or tertiary prevention, as the boundaries are sometimes blurred [49].

The intervention consists of four home visits over the span of 1 year by APNs. Prior to the visits, a social space analysis [50] is carried out for each of the seven Hamburg districts by the APNs to map out the existing service structure and identify gaps or accumulations. This will serve as a starting point for networking with the service providers as well as an orientation point for being able to refer the clients to suitable service providers with regard to the brokering and gatekeeping element of case management. Additionally, the analysis shows the need to conceptualize further consultation interventions, which have to be offered by the APNs themselves. For each district 1–2 APNs will be employed depending on the size of the population in the respective district.

The first home visits consist of the building of a professional relationship between the clients, their caregivers, and the APNs as well as a first screening of existing and potential health problems and resources. They are going to employ biographic interviews to explore important contextual information [51]. The language level will vary from basic to advanced depending on the client’s abilities. Rules of cooperation are agreed upon, and organizational and time-related issues are resolved. This is especially important from a study operations perspective. Participants in studies can sometimes get confused with the evaluation part of the study and the interventional part. It is helpful to explain the different parties and their roles in the study.

The second visit also takes place within the first month of participation and consists of a detailed assessment of health problems based on the InterRAI intellectual disability assessment [52]. A network map will be drawn to illustrate the social network of the participants. Further differentiated assessments will be used on a case-by-case level. In the aftermath, nursing problems and goals will be structured according to the ABEDL-model [53], and a case description will be prepared.

The third visit takes place in the second month and aims for the development of a prevention plan. In the prevention plan individual goals, (possible) health problems, preventive interventions, and actions to improve health as well as suitable outcomes to measure for evaluation will be formulated on the basis of the aforementioned assessments. The plan will be developed in a joint work between the client, the primary caregivers, and the APN. The fourth visit will happen after 1 year and is supposed to serve for the evaluation of the actions from the prevention plan. Moreover, overarching goals for the client's health beyond the project should be defined together with the clients and their caregivers.

Between the home visits the APNs hold liaison with the clients via their preferred ways, e.g., telephone, e-mail, or video conference. Every 2 months, the prevention plan is going to be reviewed and updated if necessary. Since it is common that people with intellectual disabilities have problems with enrolling in healthcare programs, the APNs will exercise brokering and advocacy to support them in getting the care they need. They will also gatekeep on certain interventions, if those interventions are not primarily needed. The APNs will perform single person interventions in form of counseling regarding the four areas of prevention by themselves, e.g., counseling on stress reduction if there are no suitable interventions available but will of course focus on brokering to keep it manageable. After 6 months, an individual video will be sent to the client, which focuses on motivation and resilience to keep the clients on track of their prevention plan or prompt contacts, if something is not going according to plan.

The APNs document their work in a standardized documentation system.

The CG will receive usual care. The topic of prevention and health promotion is not well covered by the existing practice and therefore we opted not to offer an active intervention. The APNs will not initiate any interaction with participants from the CG. However, because the APNs are situated right within the seven districts in offices open to the public, it cannot be excluded that control patients could receive advice or counseling on healthcare problems.

To improve adherence to the intervention, detailed intervention descriptions were developed for each home visit. However, due to the nature of this communicative, educational, coordinating complex intervention, a degree of flexibility is needed to tailor the intervention to the needs of individuals in this diverse population.

### Outcomes and data collection

Overall, the study consists of six time points (*t*_0_ to *t*_5_) and is illustrated in Table 1. The outcome evaluation will take place from *t*_0_ to *t*_2_ after 12 months. The process evaluation will take place at *t*_3_ after 13 months in the IG. We chose the additional timepoint, because simultaneous collection of outcome and process data would have compromised the rater’s blinding. The process evaluation in the waiting list group will take place at *t*_4_ after 18 months (6 months after receiving the intervention) and *t*_5_ after 24 months (12 months after receiving the intervention). The process evaluation is conducted with all participants and their primary caregivers.

The primary outcome of the study is the health status after 12 months (*t*_2_) measured with the 36-item WHODAS 2.0 interviewer version [54] (proxy). Secondary outcomes are:

- Health status after 6 months (*t*_1_) measured with 36-item WHODAS 2.0 (proxy)
- Resilience after 6 (*t*_1_) and 12 (*t*_2_) months measured with RS-11 [55] (self-assessed)
- Health-related quality of life after 6 (*t*_1_) and 12 (*t*_2_) months measured with the EQ5D-VAS [56] (proxy and self-assessed)

We will use the German version for all outcome measures.

The raters all have a background in nursing or social pedagogy. They will be trained in structured interviewing of people with intellectual disabilities as well as the respective assessments used. A pretest has been performed.

Data collection for outcome evaluation is planned as face-to-face interviews based on a questionnaire consisting of the aforementioned outcome measures as well as date of birth and data collection, sex, living situation, support through formal or informal care, and the rater’s assessment of the potential study group assignment of the participants including justification to check the blinding procedure. Data collection will be conducted in the participants’ homes or a place of their choice. This can be, for example, their own flat, a residential living group, or a residential facility. A primary caregiver (legal representative, family member or professional caregiver) is going to be present for the evaluation of the health status as well as the quality of life. This usually has the beneficial side effect that many participants feel more comfortable with their caregivers present and they can help them in clarifying questions, because they are familiar with their context, e.g., if you want to know whether the person is able to walk one kilometer the concept of 1 km is sometimes difficult to grasp. It is easier, if a caregiver can give an example of something that is as far away, e.g., a supermarket.

Data collection for the process evaluation is also planned as face-to-face interviews and is directed towards the clients as well as their primary caregivers. The questionnaire comprises of questions on the number of contacts, quality of communication between the APN and clients and caregivers, quality of collaboration, the professional relationship, satisfaction with the intervention, if new knowledge has been gained through the intervention on stress, mobility, nutrition, and addiction, and finally potential for improvement of the intervention. In our previous study on medication management of people with intellectual disabilities, it has proven useful to first ask dichotomous questions, e.g., “were you satisfied with the caregivers?” and then to ask on an ordinal scale how (un-)satisfied they were. We will employ this approach throughout the questionnaire.

We will promote participant retention through several steps:

1. Personal telephone contact with participants for scheduling data collection and intervention visits
2. Data collection reminders a week before data collection via cell phone text service
3. Intervention reminders a week before the visit via cell phone text service
4. Possibility of short-term appointment postponements (if within the time frame of data collection and intervention)

We will not collect any further outcome data from the individuals after study dropout. After discontinuation of the intervention, e.g., due to the participant’s request, we will continue to collect all outcome data up to *t*_3_.

### Data processing and analysis

Data will be transferred from the questionnaires to a prespecified data matrix in IBM SPSS Version 27 by two raters simultaneously based on a codebook of variables and their values. Data will be checked periodically by the project lead on double entries, wrong values, and missing questionnaires throughout the process to ensure data quality.

All outcomes can be analyzed with methods for continuous outcomes. We will perform an ANCOVA with the respective outcome at the respective time point (*t*_1_, *t*_2_) as the dependent variable and the baseline value (*t*_0_) as the covariate. We are going to test three hypotheses with a Bonferroni adjusted alpha of 0.025 each:

The IG differs from the CG regarding:

1. The health status
2. The health-related quality of life
3. Resilience

We will use intention-to-treat analysis for people dropping out of the study, if we have any data on them as well as participants discontinuing the intervention.

The process evaluation will be analyzed with descriptive statistics, reporting absolute and relative frequencies as well as measures of central tendency and dispersion suitable for the respective data level of the items.

Sample size calculation has been performed with the software G*power 3.1 [57]. Based on an effect size of *f* = 0.23 for an ANCOVA, a significance level of 0.05 with Bonferroni correction to 0.025, and a power of 0.8, 184 participants have to be recruited. As a conservative decision, we estimated a 30% dropout rate due to the complexity of the intervention and the 1-year time frame as well as the specifics of the target population. Therefore, we plan to include 256 people with intellectual disabilities (128 per group) in this study.

### Validity and reliability

To ensure data quality, we will hold regular appointments to discuss the process of data collection and problems during its process. We have developed a data collection manual with detailed explanation of every question in the assessments and phrasings that should explicitly not be used, because they could compromise the assessment. No external data audit is planned.

Regarding the intervention, the coordinators of the APNs will supervise them and also hold regular meetings to ensure process quality of the intervention and help in dissolving barriers. Especially in the beginning phase of the intervention, problems will inevitably come up. Since the APNs are all beginners in their new role, we believe some guidance is warranted.

### Ethics and dissemination

We do not expect any adverse health outcomes during this study, which are directly related to the intervention. Possibly, participants might feel stressed by the intervention and data collection. Therefore, we evaluate ongoing consent at every personal contact with the study or practice team. We will seek consultation with the primary caregivers in case we have the impression that something is awry, e.g., the participant does not want to participate anymore, but is too polite or hesitant to voice his feelings. Consequently, criteria for discontinuing the intervention are participants’ request or worsening of condition. We will further document reasons for dropping out of the study and inspect every case individually for relation to any harms. Additionally, the APNs are encouraged to report any adverse outcomes or errors related to their practice. After each case, the project lead will decide upon a continuation of the study. Since there is no anticipated harm, there is no compensation for trial participation.

There will be no formal data monitoring committee; however, we will present our data at the bi-yearly meetings of the project advisory board, which comprises of independent experts.

All physical data will be stored in a lockable cabinet in a lockable office. The consent forms will be archived separately. The electronic data will be stored in a password protected folder. Only the study personnel has access to this folder. The reference list with pseudonyms and plain names is stored separately by the person performing the randomization at BUAS.

The findings will be disseminated in peer-reviewed journals and presented at national and international conferences relevant to the subject fields.

### Roles and responsibilities

This is an investigator-sponsored study. Hamburg University of Applied Sciences (HUAS) is the primary sponsor with project lead Prof. Dr. Miriam Tariba Richter: MiriamTariba.Richter@haw-hamburg.de. The primary sponsor is part of the study team as consortium lead and therefore responsible for the coordination and management of the project. Recruitment of participants is the responsibility of the ESA with support by HUAS. Data collection and analysis will be performed at the BUAS. The economic analysis is carried out by the German Hospital Institute. Intervention delivery is carried out by the ESA. An independent advisory board consisting of experts from science, politics, professional practice, and the target group is going to oversee the project in two meetings each year.

### Plans for communicating important protocol amendments to relevant parties

Any modifications to the protocol that could affect the conduct of the study and the potential benefit to participants or affect participant safety, including changes in study objectives, study design, population, sample size, study procedures, or significant administrative aspects, will require a formal amendment to the protocol. We will report important changes to the protocol to the funder and need its approval as well as that of the ethics committee. In addition, the details in the German Clinical Trials Register will be revised accordingly.

## Discussion

Germany is significantly behind regarding international developments of nursing care especially in ANP. Here, distortions in the execution of the intervention may occur. For example, it will be a challenge to operate in the field of prevention and health promotion in Germany. There are a lot of barriers with regard to interprofessional collaboration due to the not well-known field of ANP. They need to show determination and perseverance to influence existing structures. Additionally, in many cases, the APNs will have to exercise a lot of advocacy to get people into existing programs.

The COVID-19 pandemic could interfere with the trial process. If there is another peak of the pandemic in late fall or winter, it could be difficult to carry out both home visits and data collection, especially for people living in institutions. Appropriate digital fallback options will be provided for this.

### Limitations

This study is only performed in the city of Hamburg. Therefore, we cannot exclude that especially in the more rural areas of Germany the intervention might be more difficult to perform. However, we believe that it should also work in other areas than Hamburg, since there might be even more need for case management and nursing expertise, because there is less availability of healthcare professionals in general.

Due to prior experiences with recruitment, we did not opt for a cluster-randomized trial. It is quite difficult to recruit a sufficient number of participants from one, e.g., institution to fill suitable clusters. Therefore, we cannot exclude possible effects of the institution on the outcomes. As mentioned before, there are no suitable assessments for health literacy for this specific topic and population and therefore we opted not to measure it with an objective outcome measure. We will measure elements of knowledge via the process evaluation in the waiting group, though.

### Trial status

This is protocol version 1.4. The first participant was recruited on 1 September 2022. Recruitment will be concluded in January 2023. The study will be completed in December 2024.

## Data Availability

The datasets analyzed during the current study and statistical codes will be available from the corresponding author on reasonable request, as is the full protocol.

## Abbreviations

ANCOVA: Analysis of covariance
ANP: Advanced nursing practice
APN: Advanced practice nurse
BUAS: Bielefeld University of Applied Sciences
CG: Control group
ESA: Evangelische Stiftung Alsterdorf
HUAS: Hamburg University of Applied Sciences
IG: Intervention group
RCT: Randomized-controlled trial

## Footnotes

1 We acknowledge that there are cultural differences in “labeling” the target population. We chose to call the population people with intellectual disabilities for this study because this comes closest to the term used in the German Social Code. We are also aware that people from the target population in Germany rather want to be called “people with learning difficulties” However, due to the adherence to the German Social Code and our funding, we use people with intellectual disabilities throughout this publication, while using “people with learning difficulties” in the day-to-day interactions.
